# Radiographic results after plaster cast fixation for 10 days versus 1 month in reduced distal radius fractures: a prospective randomised study

**DOI:** 10.1186/s13018-016-0478-7

**Published:** 2016-11-21

**Authors:** Albert Christersson, Sune Larsson, Bengt Östlund, Bengt Sandén

**Affiliations:** 1 Department of Surgical Science, Orthopaedics, Uppsala University, S-75185 Uppsala, Sweden; 2 Department of Orthopedics, Nyköping Hospital, S-61185 Nyköping, Sweden

**Keywords:** Distal radius fracture, Conservative treatment, Early mobilisation, Closed reduction, Plaster cast, Radiographic evaluation, Prospective, Randomised

## Abstract

**Background:**

The aim of this study was to examine whether reduced distal radius fractures can be treated with early mobilisation without affecting the radiographic results.

**Methods:**

In a prospective randomised study, 109 patients (mean age 65.8 (range 50–92)) with moderately displaced distal radius fractures were treated with closed reduction and plaster cast fixation for about 10 days (range 8–13 days) followed by randomisation to one of two groups: early mobilisation (*n* = 54, active group) or continued plaster cast fixation for another 3 weeks (*n* = 55, control group).

**Results:**

For three patients in the active group (6%), treatment proved unsuccessful because of severe displacement of the fracture (*n* = 2) or perceived instability (*n* = 1). From 10 days to 1 month, i.e. the only period when the treatment differed between the two groups, the active group displaced significantly more in dorsal angulation (4.5°, *p* < 0.001), radial angulation (2.0°, *p* < 0.001) and axial compression (0.5 mm, *p* = 0.01) compared with the control group. However, during the entire study period (i.e. from admission to 12 months), the active group displaced significantly more than the controls only in radial angulation (3.2°, *p* = 0.002) and axial compression (0.7 mm, *p* = 0.02).

**Conclusions:**

Early mobilisation 10 days after reduction of moderately displaced distal radius fractures resulted in both an increased number of treatment failures and increased displacement in radial angulation and axial compression as compared with the control group. Mobilisation 10 days after reduction cannot be recommended for the routine treatment of reduced distal radius fractures.

**Trial registration:**

ClinicalTrail.gov, NCT02798614. Retrospectively registered 16 June 2016.

## Background

The contribution of a plaster cast to avoid displacement after a distal radius fracture has been investigated in several studies. This research has shown that treatment with early mobilisation of non-displaced or minimally displaced distal radius fractures largely produces the same radiographic result as conventional plaster cast fixation [1–3]. When slightly displaced distal radius fractures were reduced and randomised to immobilisation in a plaster cast for 3 weeks compared with 5 weeks, early mobilisation did not lead to a greater loss of reduction in two studies [4, 5] but to a slight increase in radial angulation in one study [6]. Sarmiento introduced the conservative method of functional bracing in the 1980s [7, 8], and several subsequent studies have shown no difference in radiographic outcome between functional bracing and plaster cast fixation in moderately displaced and reduced distal radius fracture [9, 10]. Only one study has shown inferior radiographic results after early mobilisation in a functional brace compared with cast immobilisation of displaced and reduced fractures. In this study, even severely displaced fractures were included [11]. Thus, most of the studies on early mobilisation in distal radius fractures have shown that the conventional plaster cast provides very limited or no additional effect on the final displacement of reduced distal radius fractures when compared with fractures treated with less rigid fixation. A plaster cast is thought to prevent dorsal angulation but is less effective in preventing compression. It has been shown that the amount of axial compression (or ulnar variance) has a high tendency to return to the pre-reduced position after reduction and treatment in a plaster cast [12–15]. The capability of a plaster cast to retain the position in a reduced distal radius fracture also depends on the age of the patient. The older the patient, the more the fracture will redisplace when treated in a plaster cast, which is due to inferior bone quality with advanced age [12, 14, 16, 17]. There seems to be a dividing line around 45–65 years of age after which fracture instability during conservative treatment in a plaster cast increases substantially [16, 18–20]. The influence of persisting deformity on clinical outcome has been controversial for many years. However, the most established current opinion is that there is a connection between the final radiographic deformity and the remaining clinical disability after a distal radius fracture [21]. In young patients, final dorsal angulation >10–15°, radial angulation (or radial inclination) <10–15° or axial compression >2 mm are likely to give poorer clinical results [22–28]. Even in this matter, there is a difference between elderly and young people. In dependent elderly patients, the association between clinical and radiological results is much weaker and these patients seem to do well despite pronounced final deformity [27, 29–34].

An unanswered question about conservative treatment of reduced distal radius fractures is whether the maintenance of the reduction depends on the support provided by the plaster cast or by the fracture itself.

The aim of this study was to compare the differences in radiographic displacement between plaster cast fixation for 10 days compared with fixation for 1 month after reduction in moderately displaced distal radius fractures. The hypothesis was that redisplacement during the course of healing depends more on the stability of the fracture itself than on the additional stability provided by the plaster cast.

## Methods

We performed a randomised prospective study from September 2002 to January 2010 at Uppsala University Hospital in which all patients who underwent closed reduction and plaster cast fixation of a dorsally angulated distal radius fracture (Colles’ fractures) were screened for inclusion. To purify the effect of the plaster cast and minimise the stabilising effect of the fracture fragments, only patients >50 years of age were included. The ordinary protocol for the acute treatment of displaced distal radius fractures at our clinic was used during the study. The fractures were manually reduced by the on-call doctor and fixed with a splint made of plaster of Paris, covering approximately two thirds of the circumference of the dorsal aspect of the wrist and extending from below the elbow down to the metacarpophalangeal joints. Inclusion criteria were age ≥50 years, low-energy trauma, closed fracture, reduction within 3 days from injury and a previously uninjured ipsilateral and contralateral wrist. The radiographic inclusion criteria for the fractures were based on the primary dislocation: moderate dorsal angulation 5–40° from a line perpendicular to the long axis of the radius, axial compression ≤4 mm, intra-articular step-off ≤1 mm and intact ipsilateral ulna (except for processus styloideus ulnae). According to previous studies, fractures with slight dorsal angulation <5° can be treated with early mobilisation [1–3]. These fractures were therefore not included in the study. Fractures with severe dorsal angulation >40°, axial compression >4 mm or intra-articular step-off > 1 mm are not suitable for conservative treatment. These fractures were treated surgically and thus not included in the study. Patients with dementia or inflammatory joint disorders were not included.

The study was approved by the Ethical Committee of Uppsala University (Dnr 216-00), and informed consent was obtained from all patients according to the ethical guidelines of the Helsinki Declaration.

The randomisation was prepared by writing the two treatment options on papers and then placing the papers in an order taken from a table of random numbers generated from a computer. The papers were folded and put in sealed, numbered envelopes. It was not possible to reveal the choice of treatment without opening the envelopes. A log was kept to ensure that the envelopes were opened sequentially. The inclusion took place at the first follow-up at about 10 days (range 8–13) after reduction. A condition for inclusion was that the radiograph at this follow-up showed a persistent acceptable position of the fracture defined as dorsal angulation <25° and axial compression <4 mm. A fracture with larger displacement than this can cause residual disability [21, 22, 24, 28, 35], and these fractures, which were not included in the study, underwent operative treatment. This procedure ensured that unstable fractures, not suitable for conservative treatment, were excluded from the study.

**Fig. 1:**
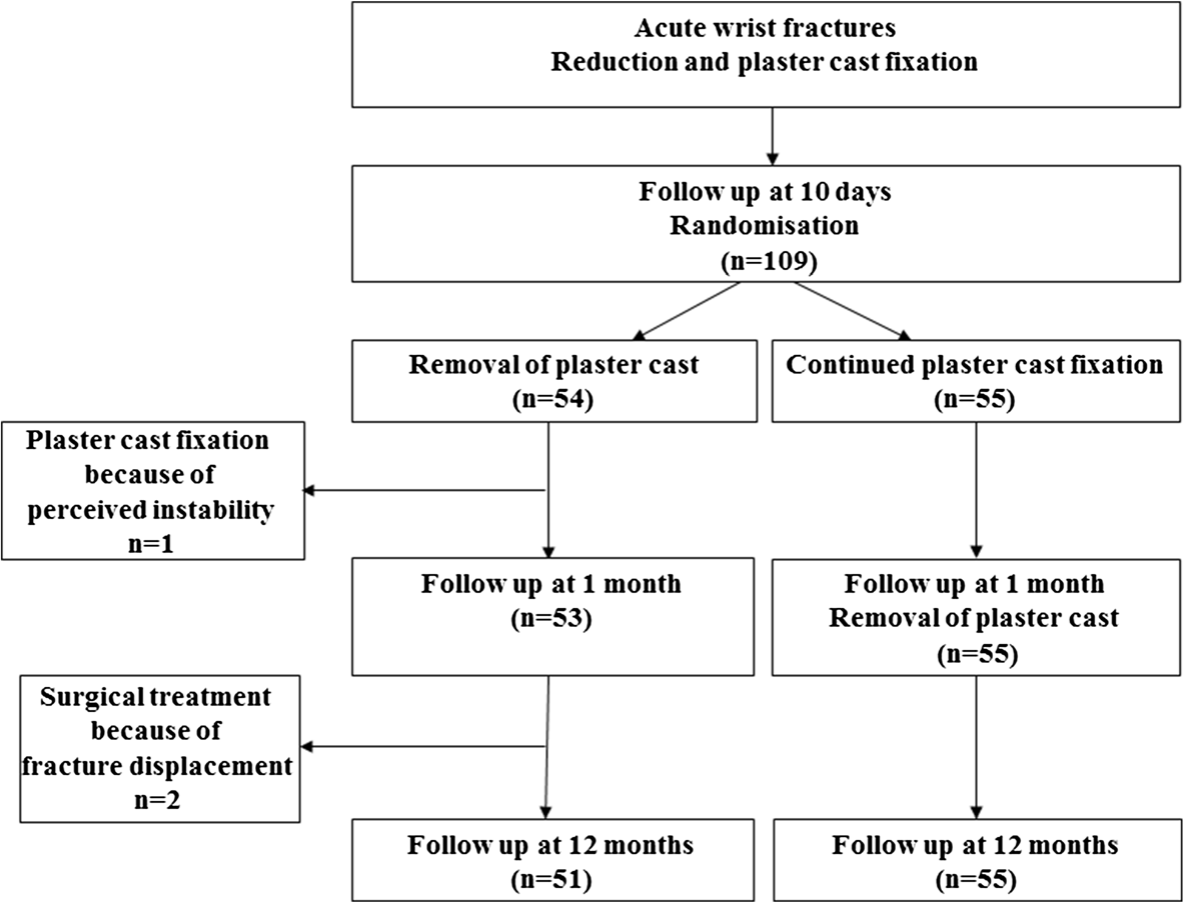
Flow diagram

**Fig. 2:**
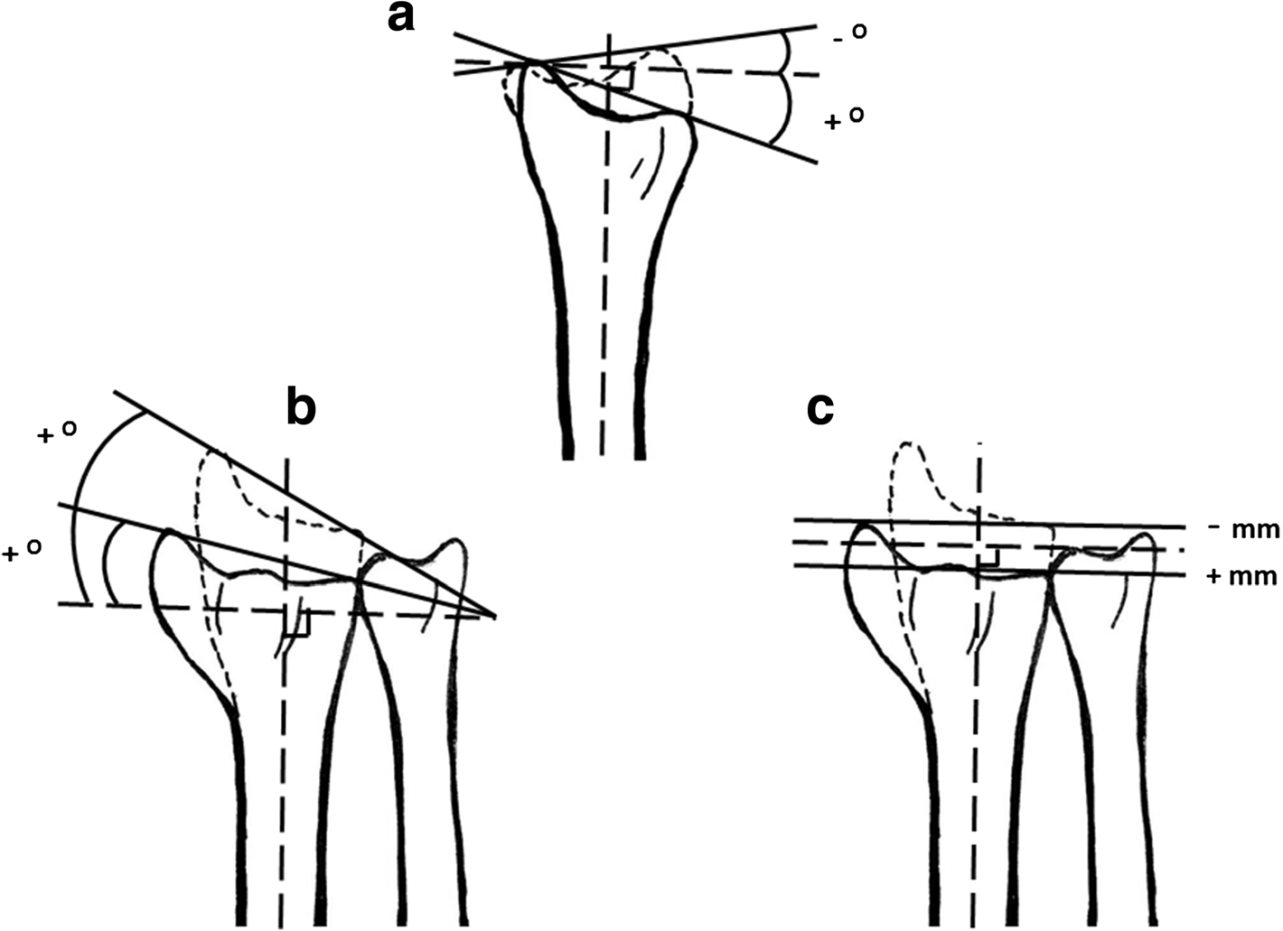
**a** Dorsal angulation was measured on the lateral view as the angle between a line connecting the anterior and posterior edge of the distal joint line of radius and a line perpendicular to the long axis of radius. *Negative values* denote volar angulation whilst *positive values* refer to dorsal angulation in relation to the line perpendicular to the long axis. The mean value of the uninjured contralateral wrists was −6.9°. **b** Radial angulation (or radial inclination) was measured on the anteroposterior view as the angle between a line connecting processus styloideus radii and the most ulnar part of the distal radius at the distal radioulnar joint (DRU joint) and a line perpendicular to the long axis of the radius. The mean value of the uninjured contralateral wrists was 21.3°. **c** Axial compression (or ulnar variance) was measured on the anteroposterior view as the distance between the distal joint line of the radius at the DRU joint and the most distal surface of the caput ulnae along the long axis of the radius. *Negative values* denote radius being longer than ulna, whilst *positive values* refer to radius being shorter than ulna. The mean value of the uninjured contralateral wrists was −1.3 mm

In total 109 patients were included. Fifty-four patients were randomised to immediate removal of the plaster cast (active group), and 55 patients were randomised to continued plaster cast fixation for another 3 weeks, totally 4–5 weeks after reduction (control group). The patients treated with removal of the plaster cast at the 10-day follow-up received an elastic bandage around the wrist and were instructed to move the wrist freely to the best of their ability, but to avoid painful activity and heavy weight lifting (Fig. 1). All fractures were radiographed at admission (i.e. the day of injury or in some cases the day after the injury), and at about 10 days (range 8–13 days), 1 month (range 4–5 weeks) and 12 months (range 11.5–12.5 months) after admission. Dorsal angulation, radial angulation and axial compression were measured digitally on all radiographs (Fig. 2a–c). For this purpose, a digital radiographic system (AGFA Web 1000) was used with software that calculated angulations and distances after defining joint lines and long bone axes. All radiographs at 10 days were taken with the plaster cast still on, whereas all radiographs at 1 month were taken without the plaster cast. The radiographs were therefore blinded for treatment.

The change in dorsal angulation, radial angulation and axial compression from admission to 12 months and from 10 days to 1 month, respectively, were calculated. The power calculation was based on the change in dorsal angulation from admission to 12 months. The study was designed for demonstrating a difference in dorsal angulation between the groups of 0.5 standard deviation, which is often referred to as a medium-sized standardised effect [36]. We assumed that the standard deviation for the change in dorsal angulation from admission to 12 months was 10°, and the study was powered to detect a difference between the groups of 5°, which is our estimation of a clinically relevant difference between the groups. For a 5% significance level and a power of 80%, a sample size of 63 patients in each group was needed.

### Statistics

All radiographic parameters were normally distributed according to the appearance on histograms and Shapiro Wilk’s *W* test (>95%). Means with 95% confidence intervals were computed for all the radiographic results presented on graphs, and Student’s *t* test, with a significance level of 0.05, was conducted for baseline characteristics and radiographic changes in displacement. Fisher’s exact test was used for proportions.

## Results

In all, 109 patients were included in the study (54 patients in the active group and 55 in the control group). The active and control groups were similar in age, injured side and fracture classification (Table 1).

**Table 1:** Patient baseline characteristics

Characteristic	10-day cast (active group)		1-month cast (control group)		*P* value
Number of patients (*n*)	54		55		
Gender F/M (*n*)	47/7		51/4		
Age in years, mean (range)	67.0	(52–90)	64.7	(50–92)	0.22
Injured side					
Right/left (*n*)	19/36		25/29		
Dominant/non-dominant (*n*)	26/28		23/32		
Fracture classification (AO)					
23A3/23C2/23C3 (*n*)	29/21/4		31/20/4		
Fracture dislocation at admission, mean (SD)					
Dorsal angulation, degrees	22.6	(8.2)	25.4	(8.0)	0.08
Radial angulation, degrees	15.4	(5.0)	13.7	(5.7)	0.10
Axial compression, mm	−0.2	(1.6)	0.3	(1.7)	0.14

Although not significant, there was a tendency for the fractures in the control group to be slightly more displaced at admission in dorsal angulation, radial angulation and axial compression than the patients in the active group.

In the active group, treatment was unsuccessful and had to be changed in three patients (3/54), but in the control group there were no cases of failure leading to treatment changes. In two of these unsuccessful cases, the radiographs at 1 month revealed that the fractures had severely displaced after removal of the plaster cast at 10 days. In the first patient, the fracture had displaced in dorsal angulation, and because of constant pain and inferior functioning of the wrist, the patient underwent osteotomy, reduction and volar plate fixation after the 1-month follow-up. This fracture was preoperatively the most dorsally angulated fracture in the study (42.7°). In the other patient, radial angulation was the most pronounced displacement. The patient reported increasing pain and disability, and underwent osteotomy, reduction and dorsal plate fixation 10 months after the fracture. This fracture was preoperatively the most radially angulated fracture in the study (−0.5°). Both these fractures fulfilled the inclusion criteria and were primarily successfully reduced. The third patient felt instability in the fracture area immediately after removal of the plaster cast at 10 days. The patient insisted on getting a new plaster cast and was treated with a new cast in situ for another 3 weeks. The fracture eventually healed in a good position. No other patient in the active group complained of instability after removal of the plaster cast. The characteristics of the three excluded patients were included in the baseline characteristics, but otherwise all radiographic parameters of the three treatment failures were excluded from the radiographic results.

**Fig. 3:**
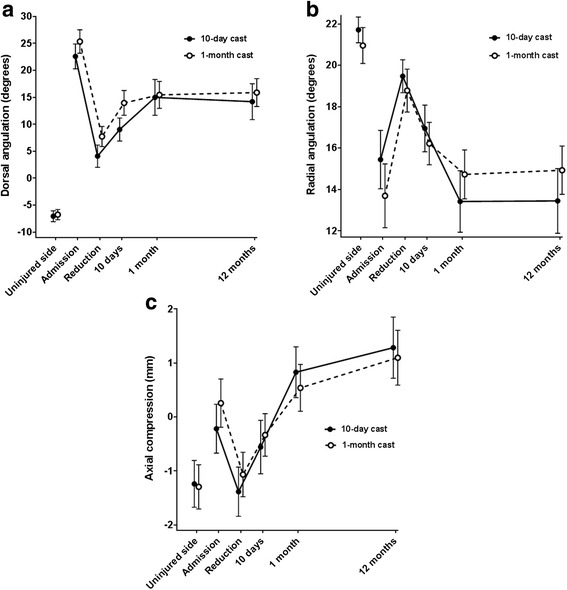
**a** Dorsal angulation, **b** radial angulation and **c** axial compression from admission to 12 months (mean with 95% confidence interval). Three failures in the 10-day cast group have been excluded

The radiographic results are presented in Fig. 3a–c. From 10 days to 1 month, i.e. the only period when the type of treatment differed between the two treatment groups, the active group redisplaced significantly more than the control group in dorsal angulation, radial angulation and axial compression. During this period, the active group redisplaced 4.5° (*p* < 0.001) more in dorsal angulation, 2.0° (*p* < 0.001) more in radial angulation and 0.5 mm (*p* = 0.01) more in axial compression compared with the control group. Seen over the entire study, i.e. from admission to 12 months, the fractures in the active group redisplaced 1.1° (*p* = 0.48) more in dorsal angulation, 3.2° (*p* = 0.002) more in radial angulation and 0.7 mm (*p* = 0.02) more in axial compression than the control group.

## Discussion

This study examined whether moderately displaced distal radius fractures treated with reduction and plaster cast fixation could be mobilised already at 10 days without an increase in redisplacement and complication rate when compared with fixation in a plaster cast for 1 month. Because fixation with a plaster cast after reduction in distal radius fractures has shown preservation of the dorsal angulation after reduction [13–15], our primary aim was to examine what happens with respect to dorsal angulation in reduced fractures when treated with early mobilisation. The question was whether a plaster cast supports a distal radius fracture directly, and if so, displacement might occur if the plaster cast were removed before the fracture has healed. Alternatively, a preserved fracture position after reduction could be contingent on the inherent stability of the fracture itself because of interference between the reduced fracture fragments and hence is not affected by early removal of the plaster cast.

The main focus in this study was the difference between the two treatment groups in radiographic displacement. However, the difference between the groups in failure rate is also an important outcome to consider. Our findings showed that treatment failure occurred in 3/54 patients (6%) in the active group (i.e. mobilisation 10 days after reduction) versus no such failures in the controls (i.e. patients treated with plaster cast for 1 month). In 2/3 failures, the patients had to undergo surgery to restore an adequate position at the fracture site whilst in one patient, a good result was achieved simply by treating the patient with a new plaster cast for an additional 3 weeks. It should be noted that the fracture in the patient who felt fracture instability immediately after early plaster cast removal was located slightly more proximal than the other fractures in the study, close to the transition between the metaphysis and the diaphysis. The anatomical location might have contributed to the feeling of instability.

From 10 days to 1 month, the fractures in the active group displaced significantly more than the fractures in the control group in dorsal angulation, radial angulation and axial compression. These findings suggest that plaster cast fixation of reduced distal radius fractures has a stabilising effect against displacement from 10 days to 1 month after reduction. If the plaster cast is removed 10 days after reduction, the fracture will displace significantly more than it would with continued fixation in a cast. However, the differences in displacement from 10 days to 1 month were small (4.5° in dorsal angulation, 2.0° in radial angulation and 0.5 mm in axial compression). Seen over the entire treatment period, i.e. from admission to 12 months, the differences between the groups decreased in dorsal angulation to 1.1°, which was no longer a significant difference, but increased in both radial angulation (3.2°) and axial compression (0.7 mm). These differences in radiographic redisplacement between the fractures in the active and control groups are small. However, the treatment should aim for reducing the residual deformity although the clinical significance of such a small redisplacement during treatment is controversial. In addition, treatment was ineffective for three of the patients in the active group, of which two patients had to undergo surgery. To motivate a failure rate of 6% (3/54 patients) for early mobilisation and an increase in redisplacement in the rest of the patients, even though the increase is small, the functional benefits after early mobilisation for the remaining patients have to be substantial. Otherwise, the regime with early mobilisation cannot be justified. When Sarmiento introduced the conservative method of functional bracing of distal radius fractures in the 80th, he never compared the outcome of his new treatment with other methods. He only stated the high value of early mobilisation from a logical perspective. Later, it was shown that functional bracing does not lead to a superior functional final result compared with conventional plaster cast fixation, but only to a transient positive effect [10, 11].

At admission, there was a tendency for slightly more displaced fractures in the control group (Table 1). Although the difference was not statistically significant, it was an unexpected finding. A thorough assessment of the randomisation procedure was therefore performed without finding any reason to believe that there was a defect in the process. We suggest that the small difference observed between the treatment groups at the time of randomisation was a random effect, as the process was carried out rigorously and systematically. It is important to note that the fractures in the control group were somewhat more displaced, but it was still the fractures in the active group that redisplaced more. It is reasonable that the more displaced a fracture is initially, the more it will redisplace in a plaster cast after reduction. Although the tendency towards a small difference in fracture displacement between the groups at admission is an unexpected limitation of the study, we still believe that the increased change in redisplacement noted in the active group is an effect of the early removal of the plaster cast.

When looking at the final radiographic position of the fractures in both the active and the control group in comparison to the deformity at admission, our results are in accordance with other studies in which conservative treatment has been shown to prevent some redisplacement in dorsal angulation after reduction, but not in radial angulation or axial compression [12, 13, 37, 38]. Both treatment groups in our study healed in a position where the dorsal angulation was better than at admission, the radial angulation was approximately the same as at admission and the axial compression was worse than at admission (Fig. 3a–c). In this perspective, it is surprising to find that dorsal angulation is the deformity that suffers the least from early mobilisation. Radial angulation in particular, but also axial compression, which historically has been considered not retainable during conservative treatment, was found to worsen significantly after early mobilisation compared with traditional plaster cast fixation for 1 month. The previous studies that reported increased deformity after early mobilisation also found that radial angulation was the deformity that redisplaced the most after early mobilisation [6, 11].

We have shown that a conventional plaster cast has a protective effect against redisplacement after reduction of a moderately displaced distal radius fracture. This protective effect was most apparent in radial angulation though it was also seen in axial compression. Further studies may help to identify subgroups of distal radius fractures in which the clinical and economic benefits of early mobilisation after reduction outweigh the risk of redisplacement. At the moment, however, early mobilisation of moderately displaced and reduced distal radius fractures cannot be safely recommended.

The sample size in the study did not reach the predetermined sample size from the power analysis. However, the numbers of patients included in the study were satisfactory for showing significant differences between the two treatment groups for all radiographic variables, except for the change in dorsal angulation from admission to 12 months, which was the variable that was used for power calculation.

## Conclusions

Early mobilisation 10 days after reduction of moderately displaced distal radius fractures resulted in both an increased number of treatment failures and increased displacement in radial angulation and axial compression as compared with the control group. Mobilisation 10 days after reduction cannot be recommended for the routine treatment of reduced distal radius fractures.
